# Artificial light at night during early development directly affects embryonic but not larval quality in a wild coral reef fish

**DOI:** 10.1093/conphys/coaf041

**Published:** 2025-06-17

**Authors:** Thibaut Roost, Jade Hargous, Lise Van Espen, Jules Schligler, Shaun S Killen, Ricardo Beldade, Stephen E Swearer, Suzanne C Mills

**Affiliations:** UAR 3278 CRIOBE, BP 1013, PSL Université Paris: EPHE-UPVD-CNRS, 98729 Papetoai, Moorea, French Polynesia; National Centre for Coasts and Climate, School of Biosciences, University of Melbourne, Royal Parade, Parkville, VIC 3010, Australia; UAR 3278 CRIOBE, BP 1013, PSL Université Paris: EPHE-UPVD-CNRS, 98729 Papetoai, Moorea, French Polynesia; Unité de Biologie des Organismes Marins et Biomimétisme, Université de Mons, 31 Boulevard Dolez, 7000 Mons, Hainaut, Belgium; UAR 3278 CRIOBE, BP 1013, PSL Université Paris: EPHE-UPVD-CNRS, 98729 Papetoai, Moorea, French Polynesia; Institute of Biodiversity, Animal Health and Comparative Medicine, College of Medical, Veterinary and Life Sciences, University of Glasgow, Graham Kerr Building, Science Way, Glasgow G12 8QQ, UK; UAR 3278 CRIOBE, BP 1013, PSL Université Paris: EPHE-UPVD-CNRS, 98729 Papetoai, Moorea, French Polynesia; Facultad de Ciencias Biologicas, Pontificia Universidad Católica de Chile, Av Bernardo O’Higgins 340, Santiago, Chile; National Centre for Coasts and Climate, School of Biosciences, University of Melbourne, Royal Parade, Parkville, VIC 3010, Australia; Oceans Institute, University of Western Australia, Fairway, Crawley 6009, WA, Australia; UAR 3278 CRIOBE, BP 1013, PSL Université Paris: EPHE-UPVD-CNRS, 98729 Papetoai, Moorea, French Polynesia; Laboratoire d’Excellence ‘CORAIL’, France; Institut Universitaire de France, Ministère de l'Enseignement supérieur, de la Recherche et de l'Innovation, 1 rue Descartes, 75231 Paris, France

**Keywords:** ALAN, cascading effects, coral reef fish, early-life traits, ecological light pollution, offspring quality, parental care, Pomacentridae

## Abstract

Artificial light at night (ALAN) is an emergent yet already global form of sensory pollution. However, its effects on marine environments remain poorly understood compared to those on terrestrial ecosystems. Low-latitude ecosystems such as shallow coral reefs might be at greater risk as they experience little change in annual day length and reef organisms rely on moonlight illumination as a zeitgeber for critical biological processes. Moreover, many coral reef fish are demersal spawners, making them vulnerable to the effects of ALAN from early life. We performed a field experiment to determine whether artificial light affects the quality of fish embryos and newly hatched larvae by exposing wild nests of the orange-fin anemonefish (*Amphiprion chrysopterus*) to white light emitting diode (LED) light (22 ± 2.0 lx; 4000 K) throughout the 6-day embryonic development period. We also explored whether light pollution indirectly influences offspring traits by measuring parental care investment. Exposure to ALAN altered embryo quality, leading to a reduction in egg volume (2.40%) and yolk reserves (6.11%) alongside an increase in heart rate (7.42%) a few hours before hatching. These changes reflect higher metabolic demands of embryos developing under light-polluted conditions. As parental care investment was unaffected by light pollution, our results suggest that these effects are more likely the consequence of a direct effect of ALAN on embryogenesis. In contrast, there was no influence of artificial light on the larval morphology or swimming performance, suggesting that the direct effects of ALAN on fish embryos do not cascade onto the larval stage immediately after hatching. These results may suggest that embryos compensated for ALAN exposure to maintain their early post-hatching larval performance. Further studies are needed to investigate whether light pollution exposure during embryonic development has delayed effects on larval performance during the dispersal phase or on larval survival.

## Introduction

Ecological light pollution originates from the use of artificial light at night (ALAN) and is a pervasive pressure threatening the nighttime environment (Longcore and Rich, 2004; Gaston *et al*., 2023). Despite artificial lighting being a symbol of modernity, security and economic development, ALAN is now widely accepted as an emergent global anthropogenic threat and one of the main causes of the decline in worldwide biodiversity (Gaston *et al*., 2021). Organisms have adapted over evolutionary time scales to predictable natural light patterns, which serve as environmental drivers for a wide array of biological rhythms (Bradshaw and Holzapfel, 2007). However, disruption of these patterns can affect molecular up to ecosystem processes (Sanders *et al*., 2021). With an estimated global annual increase of 9.6% in nighttime sky brightness over the last decade (Kyba *et al*., 2023), no equivalent pollutant over any time period has shown such a high rate of increase and thus light pollution is expected to have growing ecological impacts in both terrestrial and aquatic environments (Gaston *et al*., 2015). Indeed, shallow-water marine ecosystems are highly exposed to ALAN (Davies *et al*., 2014) with recent estimates indicating that 1.9 million km^2^ of coastal areas are exposed to biologically relevant light at night to 1 m depth (Smyth *et al*., 2021). Light pollution in marine environments has multiple origins with towns, harbours, resorts and offshore structures representing permanent light pollution sources in urbanized coastlines while shipping, fishing and recreational vessels trigger temporary ALAN (Longcore and Rich, 2004; Davies *et al*., 2014). Alongside the increasing trend in the use of artificial lighting, the world is transitioning towards more energy-efficient technologies rich in short wavelengths such as Light Emitting Diodes (LEDs). These spectra are known to be more harmful to wildlife (Zapata *et al*., 2019) and penetrate deeper in the water column (Hochberg *et al*., 2020). Consequently, this global shift in lighting technologies has the potential to further exacerbate the intensity and spatial extent of the impacts of ALAN in coastal marine environments (Davies *et al*., 2016).

Many marine organisms are likely to be affected by the introduction of artificial light as they synchronize their circadian rhythms with the predictability and duration of day–night cycles and their circalunar rhythms with natural moonlight variation throughout the lunar cycle (Marangoni *et al*., 2022). There is growing evidence that this sensory pollutant can affect a wide array of marine taxa including seagrass (Dalle Carbonare *et al*., 2023), amphipods (Navarro-Barranco and Hughes, 2015), isopods (Bullough *et al*., 2023), molluscs (Gao *et al*., 2024), sea urchins (Bari, 2024), as well as scleractinian corals (Kramer *et al*., 2023) and fish (Bassi *et al*., 2022). Indeed, light pollution represents a direct threat to coral reefs (Aubrecht *et al*., 2008) as numerous species inhabiting these ecosystems are highly photosensitive and rely on the variation in moonlight illumination for the timing of many critical biological processes such as reproduction synchronization, larval development and larval settlement (Shima *et al*., 2022; Davies *et al*., 2023). Even the light intensity of a full moon under optimal conditions (Kyba *et al*., 2017) can easily be masked by ALAN, which is typically one or two orders of magnitude brighter (Davies *et al*., 2015). Among coral reef species, teleost fish are probably the most sensitive vertebrates to extremely low light levels (Grubisic *et al*., 2019) and studying how artificial lighting affects this taxa is therefore crucial. Although evidence shows that ALAN affects fish at every level of biological organization from gene expression (Khan *et al*., 2018), physiology (Brüning *et al*., 2018), adult behaviour (Pulgar *et al*., 2019), larval settlement (O’Connor *et al*., 2019), juvenile growth and survival (Schligler *et al*., 2021), to population abundance (Bolton *et al*., 2017) and community composition (Weschke *et al*., 2024), studies on coral reef fish remain surprisingly scarce. Furthermore, >3500 coral reef fish species are demersal spawners (Jan, 2000), making them potentially more vulnerable to ALAN as reproduction and nesting can occur in shallow light-polluted waters thus exposing their offspring to artificial light from the earliest stages of life.

Given their small size and rapid development, fish embryos are particularly sensitive to disruption by environmental perturbations especially during key developmental stages called ‘windows of sensitivity’ (Agathokleous, 2022). Environmental conditions during embryonic development can result in cascading effects later in life such as changes in post-hatching survival or growth (Gagliano *et al*., 2007). This is particularly pertinent in the context of ALAN, as fish embryos can detect environmental light *in ovo* (Tamai *et al*., 2004). Indeed, the key enzyme controlling melatonin production is expressed from the first day post-fertilization in teleost fish (Gothilf *et al*., 1999; Kazimi and Cahill, 1999), which induces a functional and light-reactive circadian clock in embryos (Dekens and Whitmore, 2008). However, the effects of light pollution on fish early-life stages have been scarcely documented. Several laboratory studies have investigated the effects of artificial light on fish hatching success, a crucial step in their life cycle triggered by the onset of darkness in many reef fish (Shima *et al*., 2022). Results have been mixed with no effect on hatching (Lucon-Xiccato *et al*., 2023), delayed hatching (Duncan *et al*., 2008), reduced hatching success (Fobert *et al*., 2021), or even complete suppression (Fobert *et al*., 2019). These contrasting observations may be the result of differences in methodologies (e.g. light intensity and/or spectrum used as ALAN treatment) or species-specific sensitivities to ALAN (Brüning *et al*., 2011). On the other hand, unpublished data of a field-based study found no effect of light pollution on hatching from wild nests (Schligler *et al*., in review). Regardless, it is likely that exposure over the course of embryonic development in the wild could affect embryos prior to hatching and/or newly hatched larvae. For example, fish larvae showed increased activity and impaired cognitive abilities (Lucon-Xiccato *et al*., 2023) as well as changes in pigmentation, morphology and locomotion (Üstündağ *et al*., 2019) when embryos were exposed to light at night. There is also evidence that coral reef fish embryos developing under ALAN in a laboratory were of lower quality prior to hatching (Fobert *et al*., 2021). However, as parents were exposed prior to spawning in the latter study, the deleterious effects of artificial light on embryo quality could have been the result of a direct effect on embryonic development, an indirect parental effect on offspring quality (Gagliano and McCormick, 2009) or a combination of both. Indeed, it is known that maternal condition and parental care influence fish offspring phenotypic traits (Gagliano and McCormick, 2007; Barbasch *et al*., 2020; Cortese *et al*., 2024). ALAN may thus indirectly influence early-life stages by affecting parental traits as there is already proof of altered activity during fish paternal care provisioning (Foster *et al*., 2016).

We investigated how exposure to ALAN during embryonic development affects fish early-life stages using wild nests from a natural population of orange-fin anemonefish (*Amphiprion chrysopterus*; Cuvier, 1830) in the lagoon of Mo’orea, French Polynesia. Anemonefish are model organisms for studies on coral reef fish (Laudet and Ravasi, 2022) and their reliance on lunar light cues for reproduction makes them particularly relevant in the context of ALAN studies. By implementing a multiple Before-After-Control-Impact design, we predicted that exposure to biologically relevant levels of ALAN using custom-built underwater white LEDs on wild *A. chrysopterus* embryos during their development would (1) reduce egg volume as well as yolk sac area and affect heart rate a few hours prior to hatching. Indeed, these three metrics provide a valuable estimation of an embryo’s normal development and survival potential (i.e. embryo quality) as they are, respectively, proxies of gas diffusion efficiency (Bonisławska *et al*., 2001), available energetic reserves (Jaworski and Kamler, 2002) and physiological fitness (Perrichon *et al*., 2017). Reduced egg volume and yolk reserves under ALAN would reflect a higher embryonic metabolism over their development, with the latter being a direct consequence of increased energy mobilization and the former enabling increased oxygen supply to meet higher metabolic needs. On the other hand, embryonic heart rate could either increase or decrease with light pollution, respectively, because of stress response or as a strategy to save energy. We then tested for cascading effects of this stressor on newly hatched larvae from the embryos that developed under ALAN in the wild by measuring larval morphology and swimming performance as indicators of larval quality since these measures are correlated with survival and dispersal distance (Peck *et al*., 2012; Nanninga and Manica, 2018; Downie *et al*., 2020). We hypothesized that ALAN would (2) impair newly hatched larval quality by modifying their overall morphology and reducing their swimming capacities. Finally, we measured parental care investment of breeding pairs as a potential indirect driving mechanism for the effects observed on their offspring. In anemonefish, parental care starts immediately after the eggs are laid and increases over the next 5 days of embryonic development, peaking a few hours before hatching. This increased care may help in synchronizing hatching (Barbasch *et al*., 2022). While there is no evidence of parental investment being directly related to the quality of their embryos, the existence of a correlation between parental care and the number of offspring produced (Barbasch *et al*., 2020) implicitly suggests such a relationship. Our prediction was that ALAN would (3) not affect the proportion of time breeding pairs spend taking care of their nest a few hours before hatching. In the absence of an effect of light pollution on parental care, any influence of ALAN on embryonic and/or larval traits would thus result from a direct effect of ALAN on fish embryonic development. Indeed, indirect effects of ALAN through altered maternal condition during oogenesis (Sopinka *et al*., 2014) have been avoided in our study design as adult fish were not exposed to light pollution prior to spawning.

## Materials and Methods

### Study site and spawning monitoring

We surveyed 19 wild breeding pairs of orange-fin anemonefish (*A. chrysopterus*) in the lagoon on the north coast of the island of Mo’orea, French Polynesia (17°32′19.8” S, 149°49′46.3” W), between January and March 2023. Mature female anemonefish lay benthic eggs, one to three times per lunar cycle, and hatching occurs after sunset on the sixth or seventh night after spawning (Beldade *et al*., 2022). Eggs are attached onto the substratum under the tentacles of the magnificent sea anemone, *Radianthus magnifica* (Quoy & Gaimard, 1833), with which anemonefish adults live in an obligate mutualism.

Breeding pairs were visited every day during the week preceding and following the full moon and every 2 days during the rest of the lunar cycle to monitor their spawning. Egg clutches were identified by adult parental care behaviours (Barbasch *et al*., 2022) and confirmed by gently raising the tentacles of the anemone to locate nests. When a nest was discovered, a trained observer (T.R.) aged the eggs directly in the field and a photograph was taken to confirm ageing (see Fig. 13.1 in Beldade *et al*., 2022). Egg ageing was used to determine when to deploy the treatments and presumed hatching date (see following ‘Experimental design’ and ‘Egg sampling in the wild and hatching in the laboratory’ sections).

### Experimental design

**Figure 1:**
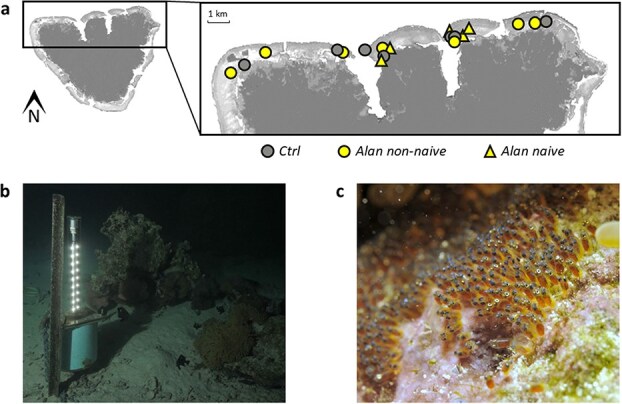
(**a**) Location of the anemonefish breeding pairs used for the study on the north coast of Mo’orea, French Polynesia. (**b**) Photograph of a custom-built underwater light system at night 1–1.5 m from an anemonefish nest (Frederic Zuberer). (**c**) Photograph of wild anemonefish embryos a few hours prior to hatching (Frederic Zuberer).

A multiple Before-After-Control-Impact design was implemented on the 19 wild fish breeding pairs. They were randomly allocated to control (‘Ctrl’; *n* = 7) or light pollution (‘Alan non-naive’; *n* = 7) treatments (Fig. 1a). To account for a potential carryover effect since ‘Alan non-naive’ pairs were previously exposed to a light pollution treatment during a study in 2021 (Schligler *et al*., in review), we included five additional anemonefish pairs naively exposed to ALAN to the experiment, i.e. with no prior exposure to ALAN (‘Alan naive’; Fig. 1a).

Regardless of the treatment, the first spawning of each breeding pair was exposed to a dummy light made from PVC pipes mimicking the structure of the underwater lights. Dummy lights were deployed attached to fence posts planted into the substrate at 1–1.5 m distance from the nest on the day of spawning when freshly spawned nests were discovered or as early as possible after spawning (Table S1). Installation of dummy lights never exceeded 10 min and any potential disturbance due to the deployment would have been the same across breeding pairs. Fish and their nest were then left undisturbed until the evening of predicted hatching when dummy lights were removed and eggs were collected for embryo quality assessment, hatchling in the lab and larval quality measurements. This first sampling represented the ‘Before’ period.

We continued to monitor breeding pairs until they spawned another time to expose this second nest to their allocated treatments (i.e. ‘After’ period) and assess offspring quality in the same conditions as the first sampling. Nests from ‘Ctrl’ pairs were exposed to the same previously described dummy light. ‘Alan non-naive’ and ‘Alan naive’ nests were exposed to light pollution by deploying custom-built underwater lights in the same way as dummy lights. Underwater lights were made from three white LED strips (4000 K) fixed to a PVC tube and supplied by a 12 V lead–acid battery, all contained within a waterproof custom-built housing (Fig. 1b). Lights were photosensitive and switched on and off automatically at dusk and dawn, respectively. They delivered a mean light intensity of 21.7 ± 1.98 lx (± SE) at 1 m distance and 4.6 ± 0.87 lx at 2 m (Table S2), which is in the range of light levels measured in near-shore light-polluted areas (5–21.6 lx, Davies *et al*., 2015; 25–68 lx, Szekeres *et al*., 2017). As anemonefish spawn one to three times per lunar cycle (Barbasch *et al*., 2020; Laudet and Ravasi, 2022), the exposure of the second nest (i.e. ‘After’ period) was carried out on the same spawning event within the lunar cycle as for the ‘Before’ period in order to control for potential variation between spawnings within the lunar cycle (e.g. if ‘Before’ exposure was during the first of two spawnings of the lunar cycle, the ‘After’ exposure was also during the first spawning of the subsequent lunar cycle). Permits for installations of underwater structures were given by ministerial decree N°2414 from the government of French Polynesia.

### Egg sampling in the wild and hatching in the laboratory

Eggs were sampled under the same conditions for both spawnings (i.e. ‘Before’ and ‘After’ periods) for every breeding pair on the predicted evening of wild nest hatching day (Fig. 1c). Sampling occurred on SCUBA following the method described in SI-2 in Cortese *et al*. (2022). Attention was taken that samples never exceeded a third of the size of the nest to limit the impact on the wild population. Each bag containing eggs collected in the wild was placed in a 40-l black-covered aquarium previously set up at 28.5 ± 0.5°C. Bags were left for at least 30 min for temperature acclimation. Following this, a random subsample of 30 eggs was collected at 6:00 pm for embryo quality measures. The remaining eggs were transferred into a hatching chamber attached to a wall of the aquarium and left to hatch overnight (see SI-2 in Cortese *et al*. (2022) for hatching protocol). A 12L:12D light cycle was provided by LED aquaria lights (Marine White Aquaray by Tropical Marine Center, Hertfordshire, UK) with lights turning on and off at 6:30 am and 6:30 pm, respectively.

### Embryo quality traits

From the subsample of 30 eggs, seven to eight were transferred into a glass petri dish filled with aquarium seawater and placed under a binocular microscope (Leica Microsystemes, Nanterre, France). Eggs showing a damaged envelope were discarded from the sample (Table S3). Using needles, eggs were gently positioned so that the embryo’s heart and yolk sac were facing upwards (Fig. S1a). Eggs were then videorecorded with a camera (Sony RX100 III) for 2 min under 30× magnification to measure embryonic heart rate. Embryos showing cardiac disturbances (e.g. irregular beating, heart stopping while filming) were not used for the analysis (Table S3). Afterwards, eggs were photographed under 12.5× magnification and pictures scaled using Leica’s software to measure egg volume and yolk sac area. The procedure was repeated until 15 embryos per nest were recorded with a regular heart rate and photographed.

The 2-min recordings were manually watched at half-speed by a single observer (T.R.) blind to treatment and period. The observer visually counted the number of heartbeats over the 2 min for each individual egg. Preliminary trials in 2020 showed no influence of the manipulation of the eggs on embryonic heart rate (*t*-test; *t* = −0.82, *df* = 12, *P* = 0.429). Photographs of the eggs were uploaded to ImageJ version 1.52 to measure egg height and width (Fig. S1a). Egg volume was then approximated as an ellipsoid (Coleman, 1991) using the formula $\mathrm{V}=\frac{4}{3}\mathrm{\pi} \ast \big(\frac{\mathrm{H}}{2}\big)\ast{\big(\frac{\mathrm{W}}{2}\big)}^{{}^2}$. V represents the volume of the egg in cubic millimetres, H the egg height in millimetres and W the egg width in millimetres. Yolk sac area was measured using the polygon selection tool on ImageJ. As every egg could not be oriented in the same position, egg rotation coded as ‘dorsal’ for dorsally positioned eggs (81.95% of total sampling; Fig. S1b), ‘tilted’ for dorso-laterally positioned eggs (9.75%; Fig. S1c) or ‘side’ for laterally positioned eggs (8.30%; Fig. S1d) was noted to correct for any effect of rotation on yolk sac area estimates.

### Larval quality traits

Larval swimming speed and morphology were measured as early as possible in the morning following the night when larvae hatched from wild-collected eggs in aquaria. We measured the maximum swimming speed (U_max_) using a constant acceleration test (Farrell, 2008) of 8–10 newly hatched larvae per nest depending on daily logistical constraints (i.e. time limitation due to the number of nests to process). Swimming tests were performed in a Blažka-type swim tunnel immersed in a larger tank (Loligo System, Viborg, Denmark) kept at a constant temperature of 28.5 ± 0.25°C. The velocity of the water flowing through the swim tunnel against which larvae had to swim was regulated by a voltmeter and we used a calibrated correspondence table to convert voltage to velocity in centimetres per second. Each larva was gently transferred into the swim tunnel and, after a 10-min acclimation period at a velocity of 1 cm.s^−1^, the constant acceleration test started and flow speed was increased by 0.5 cm.s^−1^ every 30 s until the larva could no longer maintain its position in the flow for 10 s. U_max_ was calculated according to Brett (1964): ${\mathrm{U}}_{\mathrm{max}}=\mathrm{U}+\big(\frac{\mathrm{t}}{{\mathrm{t}}_{\mathrm{i}}}\ast{\mathrm{U}}_{\mathrm{i}}\big)$. U_max_ is the maximum swimming speed in centimetres per second, U the penultimate speed in centimetres per second, U_i_ the velocity increment (here 0.5 cm.s^−1^), t the amount of time the larva was able to swim within the final increment in seconds and t_i_ the time increment (here 30 s).

After the constant acceleration test, each larva was retrieved from the swim tunnel and placed in a glass petri dish under a binocular microscope (Leica Microsystemes, Nanterre, France) and anaesthetized using MS222 to take pictures of lateral and dorsal views at 20× magnification. Pictures were subsequently uploaded to ImageJ to measure total length, standard length, body depth and body width (Fig. S2).

### Parental care investment

Prior to egg sampling in the wild, we deployed a stereo video system consisting of two GoPro HERO7 cameras permanently fixed to a metallic bar. Cameras were left for a minimum of 20 min and recordings were used to estimate parental care behaviour as well as the size of parents.

Only the recording from the camera providing the best point of view of the nest was used for the behavioural analysis. A single observer (L.V.E.) processed all the recordings blind to treatment and period. The first 7 min of each recording were considered as an acclimation period of the fish to the introduction of the cameras in their environment (Nanninga *et al*., 2017) and were therefore excluded from the analysis. Videos were then scored over 10 continuous minutes using BORIS software (Friard and Gamba, 2016). We defined six parental care behaviours to measure: fanning, mouthing, tending, chasing heterospecifics, chasing conspecifics and undetermined but on the nest (see Table S4 for definitions). The total amount of time spent displaying each of the six parental care behaviours was scored for both fish of each breeding pair. As males and females were not observed taking care of their offspring at the same time, we pooled together the durations of each parental care behaviour of each fish to obtain a total time of parental care investment per breeding pair. We expressed this amount of parental care investment as a proportion of watch time (i.e. 10 min).

To control for potential variation in egg and offspring traits due to natural differences in parental size (Green and McCormick, 2005), female and male total length of each breeding pair was estimated using the stereo video system. This required first filming a calibration panel, calculating the calibration parameters using VidSync software (Neuswanger *et al*., 2016) and then applying them to every recording. Using the same videos as for the behavioural analysis, total length of the female and male was measured and averaged three times from three different frames when fish were laterally positioned to account for measurement error. A ruler held by a snorkeler was filmed at the end of each recording, which was measured with VidSync to check the stereo video calibration. As female and male total length within pairs were significantly correlated (Pearson’s product–moment correlation; *t* = 4.34, *df* = 20, *P* < 0.001, estimate = 0.70) we decided to only use mean female total length as a covariate for statistical analyses (Table S5).

### Statistical analyses

All statistical analyses were performed in R version 4.2.3 with an alpha threshold of 0.05. To determine the effect of ALAN exposure on embryo and larval quality, we implemented linear mixed-effects regression models (LMERs) using the *lme4* package (Bates *et al*., 2015). Egg volume, yolk sac area, embryonic heart rate, larval morphology and larval maximum swimming speed were used as continuous response variables. As egg rotation had a significant effect on yolk sac area, a correction was applied on the raw values of the entire dataset according to the estimates of a linear model (Table S6). Embryonic heart rate was square root-transformed to improve linearity and normality of residuals. Larval morphology was calculated as a composite measure of total length, standard length and body depth given by the first principal component score of a Principal Component Analysis (PC1 explained 68.89% of overall variation; Table S7). Finally, U_max_ was standardized by larval total length and thus expressed in body length per second (BL.s^−1^) before being square root-transformed to improve linearity and normality of residuals.

We performed model selections for the three embryonic and the two larval quality traits using analyses of variance (ANOVAs) to compare LMERs, starting with the most complex model and sequentially dropping non-significant terms. Every model had ‘Treatment’ (categorical) in interaction with ‘Period’ (categorical) as fixed effects and site ID (i.e. breeding pair) as a random categorical effect to account for non-independence of data between periods. Depending on the response variable, mean female size (continuous) and/or egg volume (continuous) were included as fixed effects in the most complex model. Quality of each best-fit model was graphically explored using the ‘performance’ package (Lüdecke *et al*., 2021) and a correction for multiple comparison among five traits using False Discovery Rate was applied (Pike, 2011). To compare ‘Before’ periods between treatments, ‘Before’ and ‘After’ periods within treatments and ‘Before–After’ contrasts among treatments, we performed pairwise comparisons on Estimated Marginal Means (EMMs) using the *lsmeans* package (Lenth, 2020). First, we compared ‘Alan non-naive’ and ‘Alan naive’ data to test if fish offspring quality was affected by a carryover effect due to exposure to ALAN during a previous study in 2021 (Schligler *et al.*, in review). As no statistical difference was detected in the ‘Before–After’ contrasts between ‘Alan non-naive’ and ‘Alan naive’ data for four (egg volume, yolk sac area, larval morphology and maximum swimming speed) out of the five early-life stage response variables (Tables S8–S13), we considered that fish offspring quality showed no carryover effect from being previously exposed. Thus, ‘Alan non-naive’ and ‘Alan naive’ data were combined into an ‘Alan’ group and compared to the ‘Ctrl’ using the same methods as described above (Tables S14–S19).

**Figure 2:**
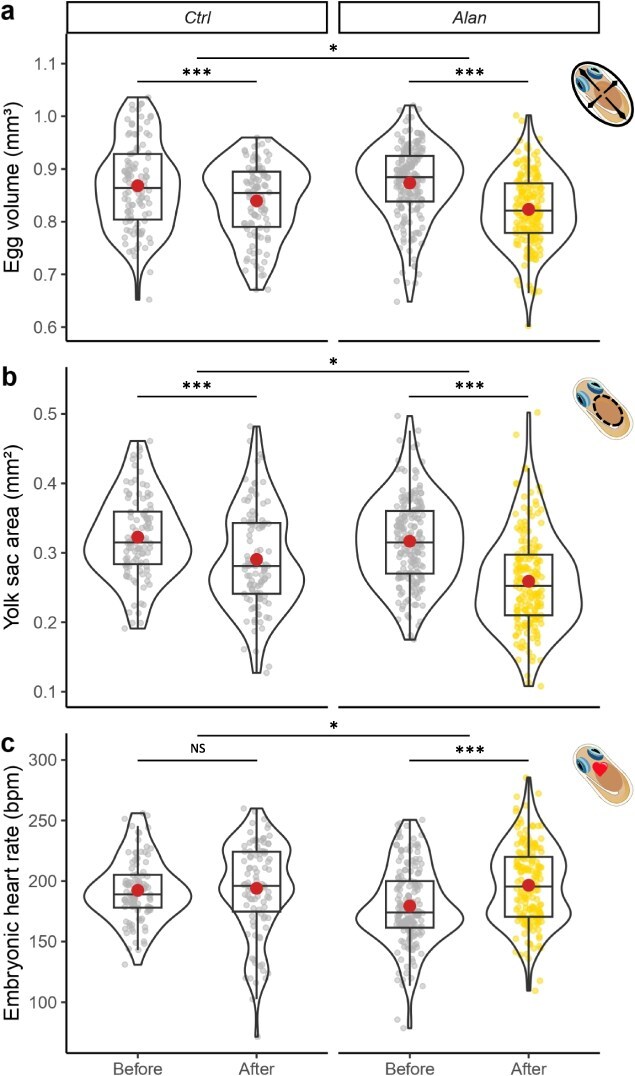
Embryonic quality traits measured prior to hatching before (first spawning) and after (second spawning) exposure of embryos to control (Ctrl) or light pollution (Alan) treatments. (**a**) Egg volume, (**b**) yolk sac area, (**c**) embryonic heart rate. Large dots represent means and smaller dots represent individual measures. Significance codes for Before–After contrasts within treatments and their differences among treatments are ^***^ for *P*-value <0.001, ^**^ for *P*-value <0.01, ^*^ for *P*-value <0.05 and NS for *P*-value >0.05.

To explore the effect of ALAN exposure on parental care investment, we implemented beta regressions on the proportion of time breeding pairs spent at parental care using the *betareg* package (Cribari-Neto and Zeileis, 2010). Fixed effects included ‘Treatment’ (categorical) in interaction with ‘Period’ (categorical). We performed pairwise comparisons on EMMs, first between ‘Alan non-naive’ and ‘Alan naive’, and subsequently between ‘Alan’ (i.e. merged ‘Alan non-naive’ and ‘Alan naive’) and ‘Ctrl’ in the same way as described above (Tables S20 and S21). There was a single recording for which no parental care has been observed due to technical issues with the camera resulting in a shorter observation time (212 s against 600). As zeros are excluded from beta regression distribution, we arbitrary attributed a value of 1 s of total parental care investment for this video before standardizing it as a proportion of watch time.

### Ethical declarations

Ethical approval for the study was granted from The Animal Ethics Committee, Centre National de la Recherche Scientifique (permit no. 006725).

## Results

### Embryo quality traits

No difference was found between ‘Before Ctrl’ and ‘Before Alan’ for the three embryo quality traits (Tables S15b, S16b and S17b), revealing a similar baseline for both treatment groups. There was a decrease in egg volume between the ‘Before’ and ‘After’ periods in both ‘Ctrl’ (EMM: estimate = 0.029 mm^3^, *t* = 3.877, *P* = 0.0001; Table S15b, Fig. 2a) and ‘Alan’ (EMM: estimate = 0.050 mm^3^, *t* = 8.713, *P* < 0.0001; Table S15b, Fig. 2a) treatments. This ‘Before–After’ contrast was, however, 72% greater in the ‘Alan’ treatment (EMM: estimate = 0.021 mm^3^, *t* = 2.260, *P* = 0.024; Table S15b) and taking into account the temporal variation estimated by the ‘Before–After’ contrast in the ‘Ctrl’ group, mean egg volume decreased by 2.40% in clutches exposed to light at night.

Similarly, yolk sac area was lower in the ‘After’ periods for both ‘Ctrl’ (EMM: estimate = 0.025 mm^2^, *t* = 3.254, *P* = 0.001, Table S16b, Fig. 2b) and ‘Alan’ (EMM: estimate = 0.044 mm^2^, *t* = 7.170, *P* < 0.0001; Table S16b, Fig. 2b) treatments. These contrasts were significantly different among treatments (EMM: estimate = 0.019 mm^2^, *t* = 2.052, *P* = 0.041; Table S16b) with ‘Before–After’ contrast being 76% greater in ‘Alan’ compared to ‘Ctrl’. As a result, mean yolk sac area was 6.11% lower for embryos exposed to artificial light at night when considering the ‘Before–After’ temporal variation estimated in the ‘Ctrl’ group.

There was no difference in embryonic heart rate between the ‘Before’ and ‘After’ periods in the ‘Ctrl’ group (EMM: estimate = −1.58 bpm, *t* = −0.385, *P* = 0.704; Table S17b, Fig. 2c) but heart rate was higher in the ‘After’ period compared to the ‘Before’ in the ‘Alan’ treatment (EMM: estimate = −14.80 bpm, *t* = −4.730, *P* = 0.0001; Table S17b, Fig. 2c). The comparison in ‘Before–After’ contrasts among treatments revealed that the contrast was 9.4 times greater in the ‘Alan’ group compared to the ‘Ctrl’ one (EMM: estimate = −13.2 bpm, *t* = 2.566, *P* = 0.018; Table S17b), revealing that mean embryonic heart rate was 7.42% greater in embryos exposed to light pollution.

### Larval quality traits

**Figure 3:**
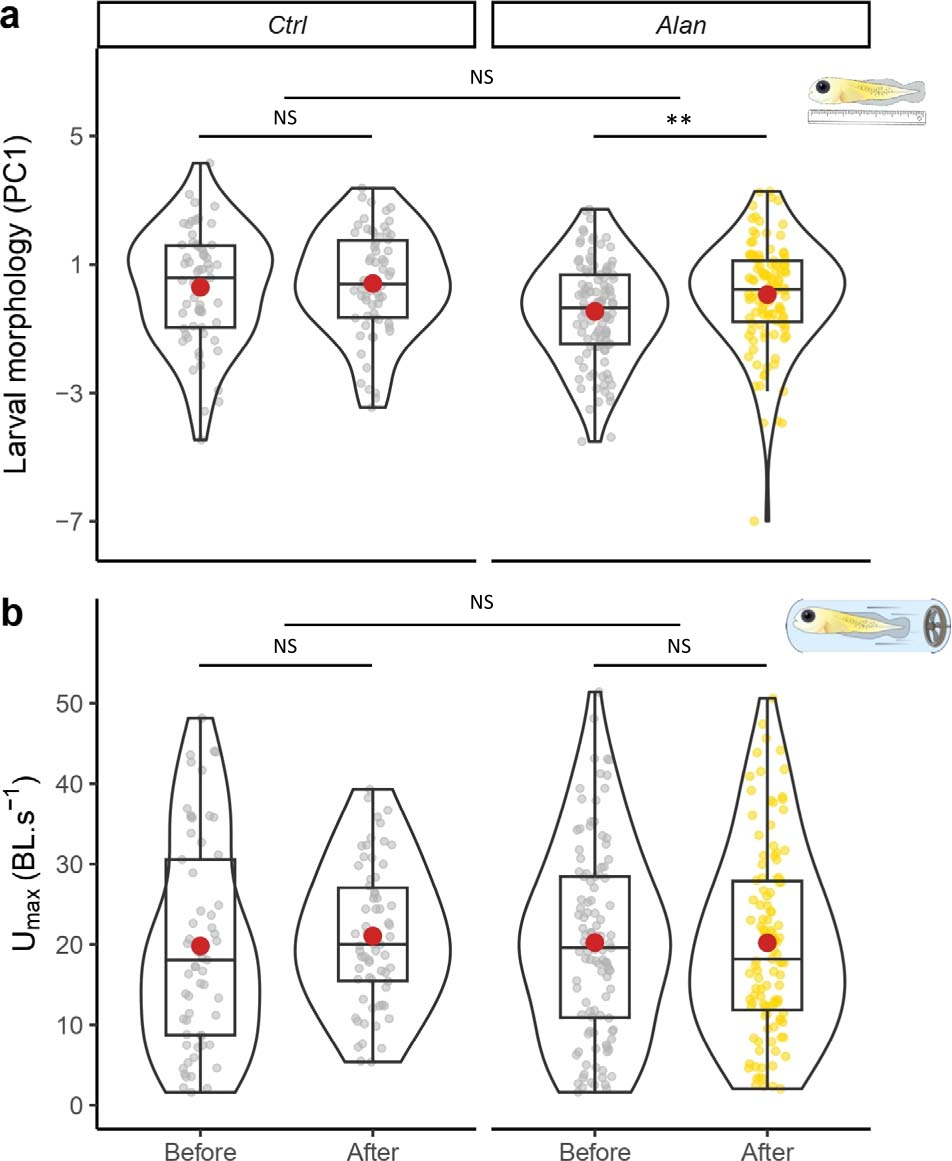
Larval quality traits measured at 0 days post-hatch before (first spawning) and after (second spawning) exposure of embryos to control (Ctrl) or light pollution (Alan) treatments. (**a**) Overall larval morphology expressed by the first principal component, (**b**) maximum swimming speed. Large dots represent means and smaller dots represent individual measures. Significance codes for Before–After contrasts within treatments and their differences among treatments are ^***^ for *P*-value <0.001, ^**^ for *P*-value <0.01, ^*^ for *P*-value <0.05 and NS for *P*-value >0.05.

No difference was found between ‘Before Ctrl’ and ‘Before Alan’ for both larval quality traits (Tables S18b and S19b), revealing a similar baseline for both treatment groups. Larvae were overall bigger in the ‘After’ period compared to the ‘Before’ period within the ‘Alan’ treatment (EMM: estimate = −0.490, *t* = −2.619, *P* = 0.009; Table S18b, Fig. 3a) but despite the absence of difference between periods within the ‘Ctrl’ group (EMM: estimate = −0.246, *t* = −0.986, *P* = 0.325; Table S18b, Fig. 3a), ‘Before–After’ contrasts were not different among treatments (EMM: estimate = −0.245, *t* = −0.786, *P* = 0.432; Table S18b).

Maximum swimming speed (U_max_) was not affected by any main effect or combination of treatment and period as suggested by the lack of difference between ‘Before’ and ‘After’ periods in the ‘Ctrl’ (EMM: estimate = −2.95, *t* = −1.558, *P* = 0.133; Table S19b, Fig. 3b) and ‘Alan’ (EMM: estimate = 0.002, *t* = 0.001, *P* = 0.999; Table S19b, Fig. 3b) treatments and in ‘Before–After’ contrast comparison among treatments (EMM: estimate = 2.95, *t* = 1.249, *P* = 0.224; Table S19b).

### Parental care

**Figure 4:**
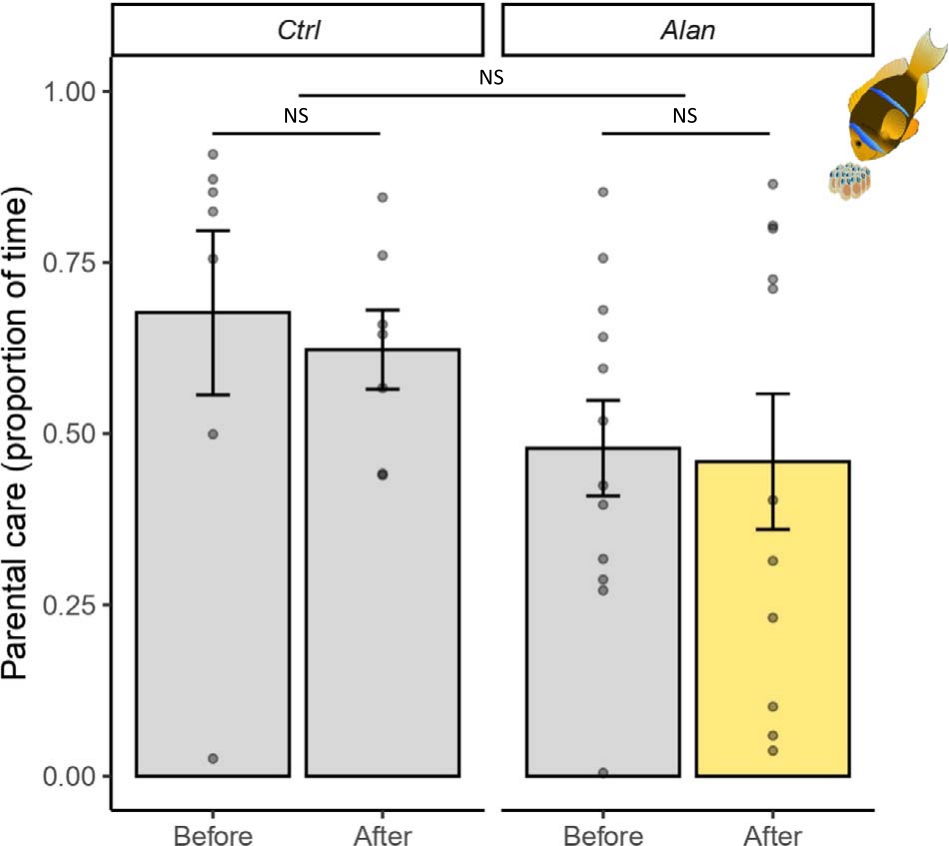
Mean proportion of time spent providing parental care for breeding pairs before (first spawning) and after (second spawning) exposure to control (Ctrl) or light pollution (Alan) treatments. Vertical error bars represent standard error of the mean, dots represent individual measures. Significance code for Before–After contrasts within treatments and their differences among treatments is NS for *P*-value >0.05.

We found significant differences between sex regarding parental care investment with females spending 4 ± 1.5% (mean ± SE) of their time displaying parental care compared to 52 ± 4.4% for males, on average. Regardless, once pooled together the proportion of time breeding pairs spent providing care to their offspring was not affected by any treatment, period or the combination of these effects (beta regression: ‘Treatment:Period’, *P* = 0.911; Table S21a). Indeed, no differences were detected between ‘Before’ periods among treatments (EMM: estimate = 0.17, *z* = 1.416, *P* = 0.157; Table S21b) ‘Before’ and ‘After’ periods for both ‘Ctrl’ (EMM: estimate = 0.02, *z* = 0.179, *P* = 0.858; Table S21b, Fig. 4) and ‘Alan’ (EMM: estimate = 0.005, *z* = 0.048, *P* = 0.962; Table S21b, Fig. 4) treatments, or between ‘Before–After’ contrasts among the two treatments (EMM: estimate = −0.02, *z* = −0.110, *P* = 0.912; Table S21b).

## Discussion

Fish early-life stages such as embryos and larvae show marked susceptibility to environmental stressors (Agathokleous, 2022). This study demonstrates that exposure to light pollution in the wild influences embryonic development of a coral reef fish. Our results show that embryos developing under ALAN had lower egg volume and yolk sac area as well as higher heart rate, suggesting higher energetic needs and a stress response. We propose that these observations are attributable to a direct effect of ALAN on embryogenesis. Indeed, we observed no influence of light pollution on parental care investment by both parents and nests were exposed only after spawning, ruling out any indirect effects of ALAN via altered parental care and/or maternal condition during oogenesis, respectively. However, further studies are needed to identify the mechanisms explaining the interaction of light pollution with embryogenesis (e.g. clock gene expression, hormone secretion). Despite the observed influence of light pollution on embryos, we surprisingly did not find any cascading effects of ALAN onto the morphology and swimming capacity of newly hatched larvae. A plausible explanation is that the ALAN exposure triggers an adaptative response in embryos, allowing them to cope with this stressor and maintain performance after hatching. However, it remains to be tested whether the metabolic costs of this response have mid- or long-term consequences such as reduced larval survival.

### Altered embryo quality as a direct effect of ALAN on development

Our study found that eggs from clutches exposed to ALAN had reduced volume, a proxy of embryo quality, as reflected in offspring survival through the juvenile stage (Baynes and Howell, 1996). Egg volume overall was also lower during the second spawning in the control treatment, likely the result of reductions in food availability (Luckenbach *et al*., 2008). The observed ALAN treatment effect was independent of this change as the comparison of contrasts between periods among treatments accounts for such variation (Fisher *et al*., 2019). Other studies have reported the same observation of reduced egg size after exposure to light pollution such as for the pond snail *Lymnaea stagnalis* (Baz *et al*., 2022) and, more interestingly, another species of anemonefish (*Amphiprion ocellaris*; Fobert *et al*., 2021). However, in these two studies parents were exposed to ALAN prior to spawning and therefore the observed reduction in egg size could be the result of an indirect parental effect, a direct effect on embryonic development or a combination of both. As eggs were only exposed to ALAN conditions after laying and no effect of ALAN was found on parental care, the reduction in *A. chrysopterus* egg volume under light pollution treatment in the present study can only be explained by a direct influence of light pollution on embryogenesis. A reduction in egg volume would modify the egg surface-to-volume ratio, which dictates gas exchange efficiency via passive oxygen supply (Bonisławska *et al*., 2001). Here, reduced egg volume would result in higher surface-to-volume ratio, thereby enhancing embryos’ passive oxygen supply to meet their higher energetic requirements under ALAN as suggested by the reduction in yolk reserves and indicative of an adaptive response.

We observed a greater impact of embryonic exposure to ALAN on an embryo’s yolk reserves, with a reduction of 6.11% after taking into account the temporal variation detected in the control group. Interestingly, Fobert *et al*. (2021) found similar results in a laboratory experiment on *A. ocellaris*. Yolk is a critical component that provides embryos with the necessary free amino acids, proteins and fatty acids for their survival and development (Jaworski and Kamler, 2002; Kamler, 2008). Yolk reserves also play a vital role in an embryo’s immune function (Zhang *et al*., 2011) and developmental timing (Schwartz *et al*., 2021). The observed reduction in remaining yolk reserve under ALAN a few hours prior to hatching might be the consequence of higher energetic needs of embryos exposed to light pollution over their development. This increase in energetic requirements could be the result of a higher metabolism at night due to light pollution, thereby increasing overall energy needs during the 5 days of embryonic development. Even though it has not been demonstrated that teleost embryos exposed to ALAN show a higher metabolism at night, natural variation in photoperiod does influence the timing of their development (Villamizar *et al*., 2013) and in birds a longer photoperiod increases embryonic metabolic rate (Cooper *et al*., 2011). While reduced remaining yolk just before hatching might not be immediately deleterious for the embryos, it could potentially affect the performance of larvae after hatching as they rely on these reserves to supply energy until first exogenous feeding (Yúfera and Darias, 2007).

Furthermore, embryos developing under ALAN showed higher heart rate (7.42%). Heart rate is known to be an indicator of physiological fitness and energetic needs in fish embryos (Perrichon *et al*., 2017), with a lower heart rate suggesting an energy-saving strategy while a higher heart rate reflects increased energy use. This metric has commonly been associated with stress response (Atherton and McCormick, 2015) and used to assess sub-lethal effects of chemicals in fish embryos (Latif *et al*., 2001; Agathokleous, 2022). Heart rate is known to increase as embryos develop (Mousavi *et al*., 2021) and we only measured it during a particular time (i.e. just upon hatching), thereby providing only a snapshot of heart rate over the developmental period. However, this estimate should reflect an integrated response of embryos to light pollution as ALAN would have affected every critical step of embryogenesis. The only other study looking at the influence of ALAN on the heart rate of a marine organism, in adult Caribbean spiny lobster (*Panulirus argus*), did not find any effect (Steell *et al*., 2020) even though the light intensity used (1 lux) was considerably lower than the present study. In a similar way as noise pollution increases embryonic heart rate in other pomacentrid species (Jain-Schlaepfer *et al*., 2018; Fakan and McCormick, 2019), our results suggest that light pollution triggers a stress response in anemonefish embryos. This is consistent on the one hand with the observed reduction in remaining yolk reserves, as stress increases energetic requirements, and on the other hand, with the decrease in egg volume, which may facilitate passive oxygen diffusion to meet elevated metabolic demand.

We estimated parental care investment by both adults within breeding pairs to understand underlying mechanisms driving the effects of light pollution on embryos. Parental care plays a crucial role in shaping offspring traits in anemonefish (Barbasch *et al*., 2022). In this study, mature adults were also exposed to ALAN, which could have modified their circadian rhythm and influenced parental care investment. However, we found no effect of ALAN on the proportion of time both adults spent providing parental care. To our knowledge no other study has investigated the influence of light pollution specifically on parental care investment, only on fish activity. ALAN increases overall activity of nesting males in smallmouth bass *Micropterus dolomieu* (Foster *et al*., 2016) as well as in bluegill *Lepomis macrochirus* (Latchem *et al*., 2021) and rockfish *Girella laevifrons* (Pulgar *et al*., 2019). On the other hand, light pollution does not affect daytime fish activity at the group level despite modifying nocturnal behaviour in another pomacentrid species, *Dascyllus aruanus* (Georgiou *et al*., 2023). Regardless, as we observed a lack of effect of ALAN on the proportion of time breeding pairs spent providing parental care, it is unlikely that light pollution affected embryo traits indirectly by modifying parental care behaviour. By exposing embryos only after they were laid, we eliminated any indirect influence of light pollution on offspring traits through modified maternal condition during oogenesis (Sopinka *et al*., 2014). This means the observed effects of ALAN on embryos can only be attributed to a direct influence of light pollution on embryogenesis. Teleost embryos are photosensitive *in ovo* and express a functional and light-responsive circadian clock early after fertilization (Tamai *et al*., 2004; Dekens and Whitmore, 2008). ALAN modifies the natural day–night cycle and thus alters melatonin secretion as demonstrated in many taxa (Sanders *et al*., 2021), which may have triggered cascading physiological effects during embryonic development. Light pollution has been shown to affect fish hatching (Duncan *et al*., 2008; Fobert *et al*., 2019, 2021), which is controlled by a hatching enzyme (McAlary and McFarland, 1993), underscoring its potential to interfere with embryonic physiology. In our study, a disruption of the embryonic circadian rhythm could have interacted with the hypothalamus–pituitary–interrenal axis, which controls stress response in fish (Sánchez-Vázquez *et al*., 2019).

### The absence of cascading effect of ALAN from embryos to larvae

Although we expected that altered embryo traits would cascade onto the larval stage, this did not occur. The lack of effect on larval morphology contrasts with the results of Üstündağ *et al*. (2019), who found that zebrafish (*Danio rerio*) larvae were smaller when embryos developed under ALAN, however their light treatment was an order of magnitude more intense (400 lux) than ecologically relevant light pollution (Davies *et al*., 2015; Szekeres *et al*., 2017). As egg size is often a good predictor of larval size in fishes (Pepin *et al*., 1997; Gisbert *et al*., 2000; Green *et al*., 2006), we expected a reduction in egg volume to result in smaller larvae. Given the effects of ALAN on embryo traits, the absence of differences in larval morphology suggests that fish embryos compensated for ALAN-induced stress by enhancing their energetic investment into growth and development, ensuring they reached a similar size at hatching. This finding is encouraging, as smaller larvae are known to have lower survival rates and reduced abilities for food acquisition and predator avoidance (Jenkins and King, 2006; Peck *et al*., 2012).

In the same vein, embryonic exposure to ALAN did not affect the swimming capabilities of newly hatched larvae. Swimming performance of teleost larvae has relevant implications for dispersal trajectories (Leis, 2007) and might be a good indicator of overall condition (Downie *et al*., 2020). As with larval morphology, the lack of influence of light pollution on larval swimming capacities suggests again that the direct effects of ALAN on embryos do not result in altered larval condition after hatching. It is worth noting that U_max_ includes both anaerobic and aerobic performances (Marras *et al*., 2010), the latter being correlated with heart rate (Clark *et al*., 2010). Therefore, the anaerobic component of U_max_ may have masked any effect of ALAN on the aerobic scope as suggested by its influence on embryonic heart rate.

Even though we found no immediate cascading effects of embryonic exposure to light pollution on early larval stage in anemonefish, evidence from other taxa suggests that delayed effects may still occur beyond hatching (Hicken *et al*., 2011). For example, toads exposed to ALAN during early life showed increased corticosterone concentration only at the juvenile stage, indicating a strongly delayed physiological response (Cope *et al*., 2020). Further studies are necessary to determine whether a similar mechanism exists in teleost fish. Additionally, measuring larval survival would provide a more holistic understanding of the cascading effects of embryonic exposure to ALAN. Given that embryos showed reduced yolk reserves it is reasonable to assume that larvae would hatch with diminished energy stores. A reduced yolk reserve shortens the critical time window for larvae to learn pre-capture skills (Yúfera and Darias, 2007) thus increasing its risk of starvation and potentially reducing larval survival, as recently demonstrated in moth (de Schoot *et al*., 2025).

## Conclusions

This study assessed the effects of ecologically relevant exposure to ALAN in the wild on embryo and newly hatched larval quality in a coral reef fish. We revealed that light pollution reduced egg volume, decreased remaining yolk reserves and increased embryonic heart rate just before hatching. Since ALAN had no detectable effect on parental care and treatments were deployed immediately after spawning to prevent any indirect maternal effects, we propose that these changes in embryo traits result from a direct effect of nighttime light exposure on the photosensitive embryos during their development. On the other hand, the absence of cascading effects on larval traits suggests that embryos may have compensated for ALAN exposure to maintain their early post-hatching performance. Pomacentrids represent >400 species, many of which are strongly site-attached and provide parental care until hatching. These results may extend to a wide range of species commonly found in shallow coral reefs, with potential implications for population dynamics and recruitment. While this study was aimed at understanding how ALAN directly affects fish embryogenesis and its eventual cascading effects onto newly hatched larvae, in natural settings site-attached parents would also be exposed to ALAN before spawning. This prolonged exposure could influence offspring traits through altered parental gametogenesis, an aspect that warrants further investigation. We call for more research on the impact of ALAN in shallow-water marine environments, as this largely unregulated stressor is expected to expand and get more intense in coastal areas.

## Supplementary Material

Web_Material_coaf041

## Data Availability

The data underlying this article will be shared on reasonable request to the corresponding author.
