# Supplementary material for: Artificial light at night during early development directly affects embryonic but not larval quality in a wild coral reef fish
Source: Conserv Physiol. 2025 Jun 17;13(1):coaf041. doi: 10.1093/conphys/coaf041 (PMC12203906; doi:10.1093/conphys/coaf041)
Supplement: Web_Material_coaf041 [file web_material_coaf041.zip › Supplementary_Material_ALAN_Clownfish_Cons_FINAL.pdf]

Supplementary material for:

Artificial light at night during early development directly affects embryonic but not larval quality in a wild coral reef fish

Thibaut Roost[1,2], Jade Hargous[1], Lise Van Espen[3], Jules Schligler[1], Shaun S. Killen[4], Ricardo Beldade[1,5], Stephen E. Swearer[2,6] and Suzanne C. Mills[1,7,8].

[1]UAR 3278 CRIOBE, BP 1013, PSL Université Paris: EPHE-UPVD-CNRS, 98729 Papetoai, Moorea, French Polynesia

[2]National Centre for Coasts and Climate and School of Biosciences, University of Melbourne, Parkville, Victoria 3010, Australia

[3]Université de Mons, Unité de Biologie des Organismes Marins et Biomimétisme, Mons, Hainaut, Belgium

[4]Institute of Biodiversity, Animal Health and Comparative Medicine, College of Medical, Veterinary and Life Sciences, University of Glasgow, Graham Kerr Building, Glasgow G12 8QQ, UK

[5]Pontificia Universidad Católica de Chile, Facultad de Ciencias Biologicas, Av Bernardo O'Higgins 340, Santiago, Chile

[6]Oceans Institute, University of Western Australia, Crawley, 6009, WA, Australia

[7]Laboratoire d'Excellence 'CORAIL', France

[8]Institut Universitaire de France

Corresponding author: thibautroost@gmail.com

Table S1. Exposition times depending on treatment and period (mean days ± SE).

	***Ctrl* (n = 7)**	***Alan non-naive* (n = 7)**	***Alan naive* (n = 5)**
Before	5.14 ± 0.14	4.71 ± 0.18	4.60 ± 0.25
After	4.71 ± 0.18	5.00 ± 0.00	4.60 ± 0.21

Table S2. Illuminance measurements (lux) taken at increasing distances from the custom-built underwater lights used to apply light at night on *Alan non-naive* and *Alan naive* nests during their *After* spawning. Illuminance was measured with a SpectroSense2+ sensor (Skye Instruments, Powys, United Kingdom) at two locations characterised by different light propagation properties: High illuminance propagation (i.e. white bottom sediment and water showing few suspended matter) and low illuminance propagation (darker bottom sediment and water showing high suspended matter). Measures were not possible at more than 5 m distance at the low illuminance propagation location due to logistical constraints.

**Location**	**Distance**	**Measure 1**	**Measure 2**	**Measure 3**
High illuminance propagation	1	25.27	25.76	27.04
	2	6.42	6.37	6.45
	3	3.57	1.93	3.19
	5	0.65	0.62	0.66
	7	0.14	0.18	0.12
	10	-0.14	-0.06	-0.26
Low illuminance propagation	1	18.45	17.85	15.80
	2	1.40	3.28	3.67
	3	0.95	0.95	0.65
	5	-0.24	-0.01	-0.04
	7			
	10			

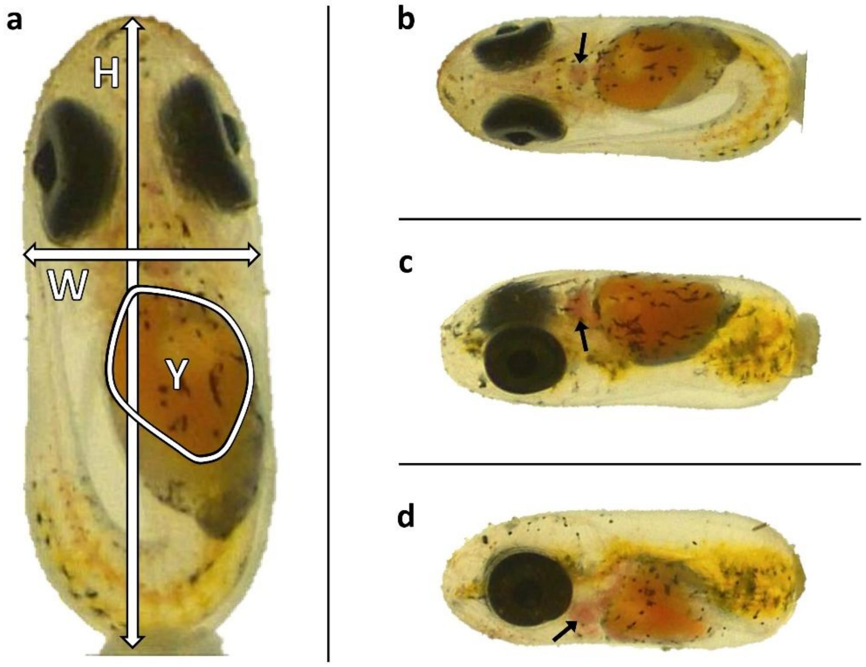

Fig. S1. (a) Representation of the measures taken on embryos to calculate egg volume (H is egg height, W is egg width) and yolk sac area (Y). Examples of embryos in *dorsal* (b), *tilted* (c), and *side* (d) rotations. Black arrows indicate the position of the heart on each rotation example.

Table S3. Number of damaged eggs and embryos showing cardiac disturbances discarded from the subsamples of eggs used for embryo quality traits measurements.

	Before			*After*		
**Site ID**	**Damaged envelope**	**Heart arrhythmia**	**Absence of heart rate**	**Damaged egg case**	**Heart arrhythmia**	**Absence of heart rate**
Rivermouth 4	1	0	0	0	0	1
ML Site	0	0	0	1	0	0
Cook Pass Shallow	2	0	0	1	0	0
Club Med Channel Shore	1	0	1	0	1	0
Opunohu Pass	3	0	0	2	0	0
PB Near	0	0	0	2	0	0
Back Row Purple	0	0	1	1	0	1
Back Row Far Right	0	0	1	0	2	0
In Front of PB	0	0	1	0	0	0
Hibiscus	1	0	2	2	1	2
Jules	0	0	2	2	0	0
Sam	1	1	1	0	0	0
Cook Dive Deep	1	1	1	2	0	0
Anne & Fred	1	0	3	0	0	0
Ben & Marianne II	0	0	0	5	0	1
Pharmacy II	2	1	0	1	0	0
Cook Dive Shallow	0	1	0	0	0	0
Megan	0	0	0	4	1	0
PB New	1	0	0	0	0	2

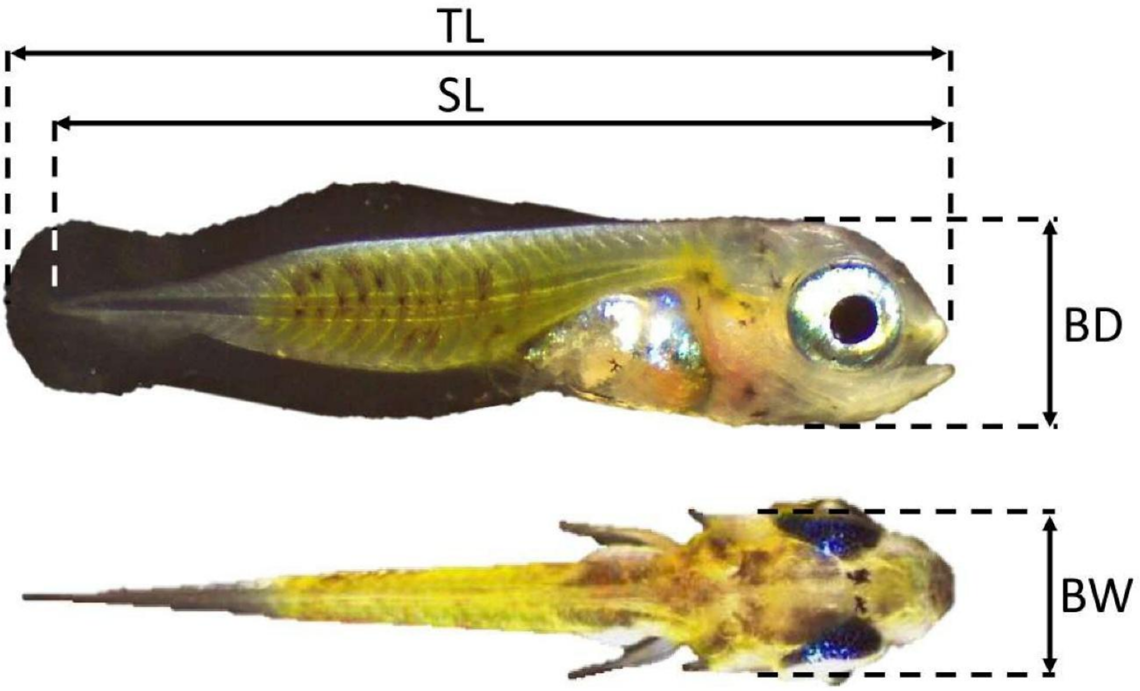

Fig. S2. Representation of larval morphology measures: Total length (TL), standard length (SL), body depth (BD), and body width (BW).

Table S4. Ethogram describing the different parental behaviours assigned to both male and female using the BORIS software.

Behaviour	Description
Fanning	Fish actively oxygenating the eggs by moving its pectoral fins on the egg clutch
Mouthing	Fish removing dead eggs or cleaning healthy eggs using its mouth
Tending	Fish is within one body length of the nest and clearly shows higher vigilance
Chasing heterospecifics	Fish chases away any fish from a different species
Chasing conspecifics	Fish chases away a fish from the same species *A. chrysopterus*
Undetermined but fish on the nest	Parental care could not be identified but the fish is less than a body length from the nest, either actively or passively

Table S5. Total length (cm) of adult females from each breeding pair measured from the stereo recordings.

Treatment	Site ID	Measure 1	Measure 2	Measure 3	Mean
Ctrl	Rivermouth 4	143.7	142.6	142.1	142.8
	ML Site	128.4	130.8	134.7	131.3
	Cook Pass Shallow	144.5	141.7	143.6	143.3
	Club Med Channel Shore	139.0	136.2	137.9	137.7
	Opunohu Pass	141.9	143.0	142.4	142.4
	PB Near	145.5	149.3	146.4	147.1
	Back Row Purple	133.3	132.5	135.3	133.7
Alan non-naive	Back Row Far Right	136.6	136.9	133.9	135.8
	In Front of PB	120.0	119.9	123.9	121.3
	Hibiscus	150.3	153.6	151.7	151.9
	Jules	117.0	118.3	120.6	118.6
	Sam	122.1	118.6	120.7	120.5
	Cook Dive Deep	135.2	133.6	136.3	135.0
	Anne & Fred	133.9	129.7	130.8	131.5
Alan naive	Ben & Marianne II	123.5	122.0	123.9	123.1
	Pharmacy II	97.3	101.6	98.1	99.0
	Cook Dive Shallow	127.4	124.1	127.5	126.3
	Megan	152.7	149.9	147.4	150.0
	PB New	148.2	151.1	151.4	150.2

Table S6. Results of the linear model (Yolk ~ Rotation + (1 | Site)) used to calculate yolk sac area corrections according to egg rotation using *Before* data (n = 211 *dorsal*, n = 36 *tilted*, n = 37 *side*). Yolk sac area correction was + 0.056 mm² and + 0.035 mm² for *side* and *tilted* embryos, respectively.

Fixed effects	Estimate	SE	df	t-value	p-value
**Intercept**	**0.319**	**0.009**	**20.149**	**36.795**	**< 2.2e-16**
**$Position_{side}$**	**-0.056**	**0.010**	**277.853**	**-5.595**	**5.28e-08**
**$Position_{tilted}$**	**-0.035**	**0.010**	**279.225**	**-3.391**	**< 0.001**

Table S7. Results from the Principal Component Analysis performed on larval morphology measurements using total length, standard length, body depth, and body width.

	**PC1 (68.89%) Inertia 2.755**		**PC2 (20.10%) Inertia 0.802**	
	Absolute contribution	Relative contribution	Absolute contribution	Relative contribution
Total length	31.90	87.90	7.12	5.71
Standard length	32.08	88.38	7.98	6.40
Body depth	25.07	69.07	0.01	0.01
Body width	10.96	30.19	84.89	68.10

Table S8. List of model selections using ANOVAs for the five response variables considering only *Alan non-naive* and *Alan naive* data. Different fixed factors were explored and tested depending on the response variable, including: **Treatment** as the experimental treatment (*Alan non-naive* vs *Alan naive*), **Period** as the periods when the sampling was performed (*Before* vs *After*), **Female** as the mean female total length of each breeding pair, **Volume** as egg volume. Model selection was performed by sequentially dropping non-significant terms to adopt the best-fit model (in bold) starting with the most complex one.

Models	AIC	BIC	LogLik	χ^2	p-value
**a) LMER: Egg volume**					
1: Volume ~ Treatment*Period + Female + (1|Site)	-976.89	-949.85	495.45		
**2: Volume ~ Treatment*Period + (1|Site)**	-977.66	-954.48	494.83	1.237	0.266
**b) LMER: Yolk sac area** *m3 is compared to m1*					
1: Yolk ~ Treatment*Period + Female + Volume + (1|Site)	-1027.02	-996.12	521.51		
2: Yolk ~ Treatment*Period + Female + (1|Site)	-994.77	-967.72	504.38	34.255	<0.001
**3: Yolk ~ Treatment*Period + Volume + (1|Site)**	-1027.80	-1000.73	520.89	1.252	0.263
**c) LMER: Embryonic heart rate**					
1: sqrt(Hr) ~ Treatment*Period + Volume + (1|Site)	953.20	979.88	-469.6		
**2: sqrt(Hr) ~ Treatment*Period + (1|Site)**	951.81	974.68	-469.9	0.606	0.436
**d) LMER: Larval morphology (1st principal component)**					
**1: PC1 ~ Treatment*Period + Female + (1|Site)**	785.42	809.24	-385.71		
2: PC1 ~ Treatment*Period + (1|Site)	792.32	812.73	-390.16	8.897	0.003
**e) LMER: Larval maximum swimming speed**					
1: sqrt(U_{max}) ~ Treatment*Period + Female + (1|Site)	761.60	785.42	-373.80		
**2: sqrt(U_{max}) ~ Treatment*Period + (1|Site)**	761.98	782.40	-374.99	2.377	0.123

Table S9a. Results of the best-fit model for egg volume from Table S8. P-values were adjusted using False Discovery Rate for five multiple comparisons. cR^2 is the conditional R^2. mR^2 is the marginal R^2, which describes the proportion of variance explained by the fixed factors alone.

Fixed effects	Estimate	SE	df	t-value	p-value	cR^2	mR^2
**Intercept**	**0.876**	**0.0178**	**11.043**	**49.235**	**<0.001**	0.450	0.109
$Treatment_{Naive}$	-0.005	0.0275	11.043	-0.177	1.00		
**$Period_{After}$**	**-0.053**	**0.0080**	**338.415**	**-6.546**	**<0.001**		
$Treatment_{Naive}$: $Period_{After}$	0.005	0.0123	338.195	0.430	1.00		

Table S9b. Post-hoc pairwise comparisons of the best-fit model for egg volume from Table S8 using Estimated Marginal Means.

Contrast	Estimate	SE	df	t.ratio	p-value
Before Alan non-naive – After Alan non-naive	**0.053**	**0.0080**	**338**	**6.545**	**<0.001**
Before Alan naive – After Alan naive	**0.047**	**0.0093**	**338**	**5.100**	**<0.001**
(Before – After Alan naive) – (Before – After Alan non-naïve)	-0.005	0.0123	338	-0.430	0.667

Table S10a. Results of the best-fit model for yolk sac area from Table S8. P-values were adjusted using False Discovery Rate for five multiple comparisons. cR^2 is the conditional R^2. mR^2 is the marginal R^2, which describes the proportion of variance explained by the fixed factors alone.

Fixed effects	Estimate	SE	df	t-value	p-value	cR^2	mR^2
Intercept	0.062	0.0462	251.052	1.334	0.305	0.489	0.231
$Treatment_{Naive}$	-0.007	0.0234	11.232	-0.301	0.769		
**$Period_{After}$**	**-0.040**	**0.0079**	**339.895**	**-5.062**	**<0.001**		
**Volume**	**0.295**	**0.0499**	**346.552**	**5.922**	**<0.001**		
$Treatment_{Naive}$: $Period_{After}$	-0.005	0.0114	337.281	-0.403	0.769		

Table S10b. Post-hoc pairwise comparisons of the best-fit model for yolk sac area from Table S8 using Estimated Marginal Means.

Contrast	Estimate	SE	df	t.ratio	p-value
Before Alan non-naive – After Alan non-naive	**0.040**	**0.0079**	**340**	**5.057**	**<0.001**
Before Alan naive – After Alan naive	**0.045**	**0.0090**	**339**	**4.994**	**<0.001**
(Before – After Alan naive) – (Before – After Alan non-naive)	0.005	0.0114	337	0.403	0.687

Table S11a. Results of the best-fit model for embryonic heart rate from Table S8. P-values were adjusted using False Discovery Rate for five multiple comparisons. Estimates are square-root transformed. cR^2 is the conditional R^2. mR^2 is the marginal R^2, which describes the proportion of variance explained by the fixed factors alone.

Fixed effects	Estimate	SE	df	t-value	p-value	cR^2	mR^2
**Intercept**	**12.840**	**0.255**	**11.479**	**50.347**	**<0.001**	0.418	0.164
**$Treatment_{Naive}$**	**1.188**	**0.395**	**11.437**	**3.009**	**0.014**		
**$Period_{After}$**	**1.047**	**0.138**	**320.892**	**7.575**	**<0.001**		
**$Treatment_{Naive}$: $Period_{After}$**	**-1.177**	**0.211**	**320.497**	**-5.572**	**<0.001**		

Table S11b. Post-hoc pairwise comparisons of the best-fit model for embryonic heart rate from Table S8 using Estimated Marginal Means. Estimates of the contrasts are given on the response scale (beats per minute).

Contrast	Estimate	SE	df	t.ratio	p-value
Before Alan non-naive – After Alan non-naive	**-27.97**	**3.74**	**11.5**	**-7.485**	**<0.001**
Before Alan naive – After Alan naive	3.63	4.46	11.4	0.814	0.432
(Before – After Alan naive) – (Before – After Alan non-naive)	**31.6**	**5.82**	**11.4**	**5.431**	**<0.001**

Table S12a. Results of the best-fit model for larval morphology from Table S8. P-values were adjusted using False Discovery Rate for five multiple comparisons. cR^2 is the conditional R^2. mR^2 is the marginal R^2, which describes the proportion of variance explained by the fixed factors alone.

Fixed effects	Estimate	SE	df	t-value	p-value	cR^2	mR^2
**Intercept**	**-6.494**	**1.9071**	**8.955**	**-3.405**	**0.030**	0.360	0.195
$Treatment_{Naive}$	0.389	0.4719	12.228	0.824	0.532		
**$Period_{After}$**	**0.543**	**0.2312**	**207.901**	**2.351**	**0.033**		
**Female**	**0.045**	**0.0144**	**8.881**	**3.150**	**0.030**		
$Treatment_{Naive}$: $Period_{After}$	-0.131	0.3634	208.031	-0.361	0.719		

Table S12b. Post-hoc pairwise comparisons of the best-fit model for larval morphology from Table S8 using Estimated Marginal Means.

Contrast	Estimate	SE	df	t.ratio	p-value
Before Alan non-naive – After Alan non-naive	**-0.543**	**0.231**	**208**	**-2.351**	**0.020**
Before Alan naive – After Alan naive	-0.412	0.280	208	-1.471	0.143
(Before – After Alan naive) – (Before – After Alan non-naive)	0.131	0.363	208	0.360	0.719

Table S13a. Results of the best-fit model for larval maximum swimming speed from Table S8. Estimates are square-root transformed. P-values were adjusted using False Discovery Rate for five multiple comparisons. cR^2 is the conditional R^2. mR^2 is the marginal R^2, which describes the proportion of variance explained by the fixed factors alone.

Fixed effects	Estimate	SE	df	t-value	p-value	cR^2	mR^2
**Intercept**	**4.000**	**0.2674**	**14.534**	**14.959**	**<0.001**	0.197	0.033
$Treatment_{Naive}$	0.722	0.4163	14.813	1.735	0.173		
$Period_{After}$	0.236	0.2208	208.188	1.070	0.357		
$Treatment_{Naive}$: $Period_{After}$	-0.583	0.3470	208.336	-1.680	0.173		

Table S13b. Post-hoc pairwise comparisons of the best-fit model for larval maximum swimming speed from Table S8 using Estimated Marginal Means. Estimates of the contrasts are given on the response scale (body length per second).

Contrast	Estimate	SE	df	t.ratio	p-value
Before Alan non-naive – After Alan non-naive	-2.0	1.82	14.4	-1.068	0.303
Before Alan naive – After Alan naive	3.2	2.44	14.8	1.290	0.217
(Before – After Alan naive) – (Before – After Alan non-naive)	5.1	3.05	14.4	1.673	0.116

Table S14. List of model selections using ANOVAs for the five response variables considering *Ctrl* and *Alan* (i.e. merged *Alan non-naive* and *Alan naive*) data. Different fixed factors were explored and tested depending on the response variable, including: **Treatment** as the experimental treatment (*Ctrl* vs *Alan*), **Period** as the periods when the sampling was performed (*Before* vs *After*), **Female** as the mean female total length of each breeding pair, **Volume** as egg volume. Model selection was performed by sequentially dropping non-significant terms to adopt the best-fit model (in bold) starting with the most complex one.

Models	AIC	BIC	LogLik	χ^2	p-value
**a) LMER: Egg volume**					
1: Volume ~ Treatment*Period + Female + (1|Site)	-1611.8	-1581.4	812.87		
**2: Volume ~ Treatment*Period + (1|Site)**	-1612.4	-1581.4	812.18	1.377	0.241
**b) LMER: Yolk sac area** *m3 is compared to m1*					
1: Yolk ~ Treatment*Period + Female + Volume + (1|Site)	-1627.6	-1593.0	821.82		
2: Yolk ~ Treatment*Period + Female + (1|Site)	-1591.4	-1561.1	802.71	38.227	<0.001
**3: Yolk ~ Treatment*Period + Volume + (1|Site)**	-1628.8	-1598.5	821.41	0.814	0.367
**c) LMER: Embryonic heart rate**					
1: sqrt(Hr) ~ Treatment*Period + Volume + (1|Site)	1608.8	1638.7	-797.38		
**2: sqrt(Hr) ~ Treatment*Period + (1|Site)**	1909.1	1634.8	-798.55	2.342	0.126
**d) LMER: Larval morphology (1st principal component)**					
**1: PC1 ~ Treatment*Period + Female + (1|Site)**	1264.2	1291.2	-625.08		
2: PC1 ~ Treatment*Period + (1|Site)	1271.2	1294.3	-629.59	9.013	0.003
**e) LMER: Larval maximum swimming speed**					
1: sqrt(U_{max}) ~ Treatment*Period + Female + (1|Site)	1170.8	1197.8	-578.4		
**2: sqrt(U_{max}) ~ Treatment*Period + (1|Site)**	1171.0	1194.1	-579.5	2.209	0.137

Table S15a. Results of the best-fit model for egg volume from Table S14. P-values were adjusted using False Discovery Rate for five multiple comparisons. cR^2 is the conditional R^2. mR^2 is the marginal R^2, which describes the proportion of variance explained by the fixed factors alone.

Fixed effects	Estimate	SE	df	t-value	p-value	cR^2	mR^2
**Intercept**	**0.868**	**0.0200**	**18.238**	**43.346**	**<0.001**	0.515	0.080
$Treatment_{Alan}$	0.005	0.0252	18.237	0.204	1.00		
**$Period_{After}$**	**-0.029**	**0.0075**	**540.029**	**-3.877**	**<0.001**		
**$Treatment_{Alan}$: $Period_{After}$**	**-0.021**	**0.0094**	**540.130**	**-2.260**	**0.040**		

Table S15b. Post-hoc pairwise comparisons of the best-fit model for egg volume from Table S14 using Estimated Marginal Means.

Contrast	Estimate	SE	df	t.ratio	p-value
Before Ctrl – Before Alan	-0.005	0.0252	18.2	-0.204	0.840
Before Ctrl – After Ctrl	**0.029**	**0.0075**	**540**	**3.877**	**<0.001**
Before Alan – After Alan	**0.050**	**0.0058**	**540**	**8.713**	**<0.001**
(Before – After Alan) – (Before – After Ctrl)	**0.021**	**0.0094**	**540**	**2.260**	**0.024**

Table S16a. Results of the best-fit model for yolk sac area from Table S14. P-values were adjusted using False Discovery Rate for five multiple comparisons. cR^2 is the conditional R^2. mR^2 is the marginal R^2, which describes the proportion of variance explained by the fixed factors alone.

Fixed effects	Estimate	SE	df	t-value	p-value	cR^2	mR^2
**Intercept**	**0.096**	**0.0392**	**320.389**	**2.457**	**0.024**	0.445	0.199
$Treatment_{Alan}$	-0.007	0.0183	19.416	-0.395	0.697		
**$Period_{After}$**	**-0.025**	**0.0075**	**540.746**	**-3.254**	**0.003**		
**Volume**	**0.260**	**0.0419**	**542.442**	**6.217**	**<0.001**		
$Treatment_{Alan}$: $Period_{After}$	-0.019	0.0094	539.832	-2.052	0.051		

Table S16b. Post-hoc pairwise comparisons of the best-fit model for yolk sac area from Table 14 using Estimated Marginal Means.

Contrast	Estimate	SE	df	t.ratio	p-value
Before Ctrl – Before Alan	0.007	0.0183	19.3	0.395	0.697
Before Ctrl – After Ctrl	**0.025**	**0.0076**	**541**	**3.254**	**0.001**
Before Alan – After Alan	**0.044**	**0.0061**	**546**	**7.170**	**<0.001**
(Before – After Alan) – (Before – After Ctrl)	**0.019**	**0.0094**	**540**	**2.052**	**0.041**

Table S17a. Results of the best-fit model for embryonic heart rate from Table S14. Estimates are square-root transformed. P-values were adjusted using False Discovery Rate for five multiple comparisons. cR^2 is the conditional R^2. mR^2 is the marginal R^2, which describes the proportion of variance explained by the fixed factors alone.

Fixed effects	Estimate	SE	df	t-value	p-value	cR^2	mR^2
**Intercept**	**13.823**	**0.258**	**20.070**	**53.592**	**<0.001**	0.294	0.040
$Treatment_{Alan}$	-0.484	0.325	20.049	-1.493	0.252		
$Period_{After}$	0.057	0.148	512.287	0.385	0.876		
**$Treatment_{Alan}$: $Period_{After}$**	**0.487**	**0.187**	**512.616**	**2.603**	**0.024**		

Table S17b. Post-hoc pairwise comparisons of the best-fit model for embryonic heart rate from Table S14 using Estimated Marginal Means. Estimates of the contrasts are given on the response scale (beats per minute).

Contrast	Estimate	SE	df	t.ratio	p-value
Before Ctrl – Before Alan	13.2	8.86	20	1.486	0.153
Before Ctrl – After Ctrl	-1.6	4.09	20.1	-0.385	0.704
Before Alan – After Alan	**-14.8**	**3.13**	**20.0**	**-4.730**	**<0.001**
(Before – After Alan) – (Before – After Ctrl)	**-13.2**	**5.15**	**20.0**	**2.566**	**0.018**

Table S18a. Results of the best-fit model for larval morphology from Table S14. P-values were adjusted using False Discovery Rate for five multiple comparisons. cR^2 is the conditional R^2. mR^2 is the marginal R^2, which describes the proportion of variance explained by the fixed factors alone.

Fixed effects	Estimate	SE	df	t-value	p-value	cR^2	mR^2
**Intercept**	**-6.191**	**2.0735**	**15.813**	**-2.986**	**0.022**	0.323	0.147
$Treatment_{Alan}$	-0.184	0.4292	21.217	-0.428	0.673		
$Period_{After}$	0.246	0.2488	329.190	3.119	0.540		
**Female**	**0.046**	**0.0147**	**15.673**	**3.119**	**0.022**		
$Treatment_{Alan}$: $Period_{After}$	0.245	0.3114	328.687	0.786	0.540		

Table S18b. Post-hoc pairwise comparisons of the best-fit model for larval morphology from Table S14 using Estimated Marginal Means.

Contrast	Estimate	SE	df	t.ratio	p-value
Before Ctrl – Before Alan	0.184	0.429	21.6	0.428	0.673
Before Ctrl – After Ctrl	-0.246	0.249	329	-0.986	0.325
Before Alan – After Alan	**-0.490**	**0.187**	**328**	**-2.619**	**0.009**
(Before – After Alan) – (Before – After Ctrl)	-0.245	0.311	329	-0.786	0.432

Table S19a. Results of the best-fit model for larval maximum swimming speed from Table S14. Estimates are square-root transformed. P-values were adjusted using False Discovery Rate for five multiple comparisons. cR^2 is the conditional R^2. mR^2 is the marginal R^2, which describes the proportion of variance explained by the fixed factors alone.

Fixed effects	Estimate	SE	df	t-value	p-value	cR^2	mR^2
**Intercept**	**4.152**	**0.2773**	**24.694**	**14.972**	**<0.001**	0.201	0.006
Treatment$_{Alan}$	0.145	0.3476	24.334	0.417	0.850		
Period$_{After}$	0.341	0.2183	329.655	1.562	0.298		
Treatment$_{Alan}$: Period$_{After}$	-0.341	0.273	329.180	-1.249	0.354		

Table S19b. Post-hoc pairwise comparisons of the best-fit model for larval maximum swimming speed from Table S14 using Estimated Marginal Means. Estimates of the contrasts are given on the response scale (body length per second).

Contrast	Estimate	SE	df	t.ratio	p-value
Before Ctrl – Before Alan	-1.23	2.92	23.6	-0.419	0.679
Before Ctrl – After Ctrl	-2.95	1.89	23.3	-1.558	0.133
Before Alan – After Alan	0.002	1.41	23.6	0.001	0.999
(Before – After Alan) – (Before – After Ctrl)	2.95	2.36	23.3	1.249	0.224

Table S20a. Results of the beta regression for the proportion of time breeding pairs spent displaying parental care considering *Alan non-naive* and *Alan naive* data. Estimates are on the logit scale.

Fixed effects	Estimate	SE	statistic	p-value	R^2
Intercept	-0.38	0.399	-0.949	0.342	0.05
$Treatment_{Naive}$	0.38	0.616	0.622	0.534	
$Period_{After}$	-0.10	0.562	-0.17	0.862	
$Treatment_{Naive}$: $Period_{After}$	0.27	0.901	-0.30	0.761	

Table S20b. Post-hoc pairwise comparisons of the beta regression for the proportion of time breeding pairs spent displaying parental care considering *Alan non-naive* and *Alan naive* data using Estimated Marginal Means. Estimates of the contrasts are given on the response scale.

Contrast	Estimate	SE	df	z.ratio	p-value
Before Alan non-naive – After Alan non-naive	0.02	0.134	Inf	0.174	0.862
Before Alan naive – After Alan naive	-0.04	0.175	Inf	-0.251	0.802
(Before – After Alan naive) – (Before – After Alan non-naive)	-0.07	0.221	Inf	-0.305	0.760

Table S21a. Results of the beta regression for the proportion of time breeding pairs spent displaying parental care considering *Ctrl* and *Alan* (i.e. merged *Alan non-naive* and *Alan naive*) data. Estimates are on the logit scale.

Fixed effects	Estimate	SE	statistic	p-value	R^2
Intercept	0.47	0.396	1.18	0.240	0.08
$Treatment_{Alan}$	-0.69	0.499	-1.38	0.167	
$Period_{After}$	-0.10	0.558	-0.18	0.858	
$Treatment_{Alan}$: $Period_{After}$	0.08	0.707	0.11	0.911	

Table S21b. Post-hoc pairwise comparisons of the beta regression for the proportion of time breeding pairs spent displaying parental care considering *Ctrl* and *Alan* (i.e. merged *Alan non-naive* and *Alan naive*) data using Estimated Marginal Means. Estimates of the contrasts are given on the response scale.

Contrast	Estimate	SE	df	z.ratio	p-value
Before Ctrl – Before Alan	0.17	0.120	Inf	1.416	0.157
Before Ctrl – After Ctrl	0.02	0.134	Inf	0.179	0.858
Before Alan – After Alan	0.01	0.107	Inf	0.048	0.962
(Before – After Alan) – (Before – After Ctrl)	-0.02	0.171	Inf	-0.110	0.912
